# Implementation of Interventions for the Control of Typhoid Fever in Low- and Middle-Income Countries

**DOI:** 10.4269/ajtmh.18-0110

**Published:** 2018-07-25

**Authors:** Raluca Barac, Daina Als, Amruta Radhakrishnan, Michelle F. Gaffey, Zulfiqar A. Bhutta, Melanie Barwick

**Affiliations:** 1 Centre for Global Child Health, The Hospital for Sick Children, Toronto, Canada;; 2 Research Institute, The Hospital for Sick Children, Toronto, Canada;; 3 Dalla Lana School of Public Health, University of Toronto, Toronto, Canada;; 4 Center of Excellence in Women and Child Health, The Aga Khan University, Karachi, Pakistan;; 5 Department of Psychiatry, University of Toronto, Toronto, Canada

## Abstract

Past research has focused on typhoid fever surveillance with little attention to implementation methods or effectiveness of control interventions. This study purposefully sampled key informants working in public health in Chile, India, Pakistan, Bangladesh, Thailand, Vietnam, South Africa, and Nigeria to 1) scope typhoid-relevant interventions implemented between 1990 and 2015 and 2) explore contextual factors perceived to be associated with their implementation, based on the Consolidated Framework for Implementation Research (CFIR). We used a mixed methods design and collected quantitative data (CFIR questionnaire) and qualitative data (interviews with 34 public health experts). Interview data were analyzed using a deductive qualitative content analysis and summary descriptive statistics are provided for the CFIR data. Despite relatively few typhoid-specific interventions reportedly implemented in these countries, interventions for diarrheal disease control and regulations for food safety and food handlers were common. Most countries implemented agricultural and sewage treatment practices, yet few addressed the control of antibiotic medication. Several contextual factors were perceived to have influenced the implementation of typhoid interventions, either as enablers (e.g., economic development) or barriers (e.g., limited resources and habitual behaviors). Consolidated Framework for Implementation Research factors rated as important in the implementation of typhoid interventions were remarkably consistent across countries. The findings provide a snapshot of typhoid-relevant interventions implemented over 25 years and highlight factors associated with implementation success from the perspective of a sample of key informants. These findings can inform systematic investigations of the implementation of typhoid control interventions and contribute to a better understanding of the direct effects of implementation efforts.

## INTRODUCTION

Typhoid fever remains a significant health burden in low- and middle-income countries (LMICs)^1^ and work is ongoing to collect global longitudinal data on enteric fevers to better understand the scale of the problem. In addition, population-based data on risk factors related to safe water, adequate sanitation, appropriate personal and food hygiene, migration, and vaccination are needed to understand pathways to change with respect to disease burden and to improve priority setting for policies and actions that can prevent and control typhoid fever. Although there is evidence of significant reductions in the disease burden of typhoid across many LMICs, especially in Latin America and Southeast Asia,^2^ research to date has focused on monitoring typhoid rates with little attention to the implementation of typhoid control interventions. There are no data available on the type of control interventions that have been implemented, nor on the implementation effectiveness of these interventions. Because the distinction between intervention effectiveness (typhoid control outcomes) and implementation effectiveness (whether interventions were well implemented) is important, the relative contributions of control interventions, such as investments in clean water, sanitation and hygiene (WASH) strategies; health and immunization strategies; food safety regulations; or socioeconomic development remain unclear. Also unknown is whether rates of disease over time relate to the success or failure of control interventions or to the implementation approach, or both.^3^ To this end, the present study purposefully sampled key informants working in public health to explore a range of typhoid-relevant interventions implemented in eight countries: Chile, India, Pakistan, Bangladesh, Thailand, Vietnam, South Africa, and Nigeria.

### Implementation issues in LMICs.

Although there is no empirical evidence on the implementation of typhoid control interventions specifically, there is some evidence on the facilitators and barriers to the implementation of other interventions in the context of LMICs, including exclusive breastfeeding, Human papillomavirus vaccinations, antenatal care and maternal health, and others.^4–7^ Many implementation barriers appear to be common across countries. For instance, a secondary qualitative analysis of meeting reports and articles describing projects undertaken by Puchalski Ritchie et al.^7^ in five LMICs on three continents found a high degree of commonality for barriers across countries and clinical areas, with lack of financial, material, and human resources featuring as most prominent. By contrast, few facilitators were identified, and these varied substantially across countries and interventions.

We have limited evidence about which implementation strategies are effective for promoting practice change in LMICs and endemic contexts. There is some evidence on the effectiveness of implementation strategies to improve uptake and compliance with evidence-based clinical practice guidelines in LMICs for mental disorders and for other noncommunicable diseases. Existing literature suggests that multifaceted implementation strategies that involve an educational component may be effective for improving guideline adherence and, subsequently, clinical outcomes.^8^

We also know little about effective implementation processes in LMIC contexts. A study of guideline utilization in Uganda revealed that of 137 health sector guidelines, 83 of which were related to Millennium Development Goals, there was no framework for systematic dissemination. More than 60% of guidelines available at the central level were not available at the service delivery level, and there was no framework for systematic monitoring of use, evaluation, and review of guidelines. Also noted was suboptimal supervision that would encourage the use of guidelines, assess their utilization, and provide feedback.^8^

### Implementation framework used in the present study.

The present study was guided by a well-documented implementation framework called the Consolidated Framework for Implementation Research (CFIR).^9^ Implementation models or conceptual frameworks enhance effectiveness of interventions by helping to focus interventions on the essential processes of behavioral change, which can be quite complex. Moreover, the use of theories and frameworks in implementation research enhances interpretability of study findings and ensures that essential implementation strategies are included.^10^ Although there are a number of guiding models and frameworks for implementation, few comprehensively address the diverse array of factors associated with implementation success. The CFIR organizes these key factors into five domains (intervention characteristics, inner setting, outer setting, staff characteristics, and the implementation process) of 37 measurable constructs (see http://www.cfirguide.org/Appendix A for a list of constructs, domains, and their definitions). The contribution of the CFIR is that it allows for the comprehensive examination of a variety of contextual factors that are empirically associated with successful implementation across a variety of disciplines (e.g., global health, education, and mental health) and that may not have been taken into account in studies using controlled designs that would have “blocked” or ignored them. The Consolidated Framework for Implementation Research also enables examination of specific challenges, barriers, and facilitators identified during implementation and evaluation of programs that have the potential to modify the effect of an intervention. For instance, a study of exclusive breastfeeding implementation in Ethiopia and Mali revealed several contextual factors that were strongly related to improved exclusive breastfeeding rates in both countries^4^ including adaptation, relative advantage, complexity, needs of the target population, networking, external policies and incentives, tension for change, change agents’ and implementers’ knowledge and attitudes about the intervention, and establishing strong champions for the intervention.

In the present study, we collected quantitative data (CFIR questionnaire) and qualitative data (interviews with key informants) with the aim of exploring and describing 1) the typhoid-relevant interventions implemented between 1990 and 2015 in the aforementioned eight countries and 2) the contextual factors that shaped their implementation. The application of CFIR to interventions well supported by evidence, such as potential typhoid control interventions, allows for the examination of contextual factors perceived to be important for successful implementation of these interventions and ultimately contributes to elucidating which factors are most strongly associated with implementation success. Qualitative interviews were conducted to explore specific typhoid-relevant interventions that were implemented in each of the eight countries. The research was overseen by a global advisory committee comprising global health experts and public health country-level experts with the requisite information and access to data.

This implementation study was conducted as a complement to a larger study exploring country-specific data sets for rates of disease burden over time.^11–18^ Scoping of typhoid-relevant interventions implemented in these eight countries and contextual implementation factors perceived to be associated with decreasing typhoid rates allows for identification of commonalities and differences for these factors across contexts and has the potential to inform implementation research and practice as well as policy and priority setting about interventions to tackle the global burden of typhoid. Together with the results of our larger quantitative study,^2,11–18^ these findings could facilitate the development of an evidence-based implementation approach for the control of typhoid in LMICs.

## METHODS

### Ethics statement.

This study was approved by the Research Ethics Board at The Hospital for Sick Children, Toronto, Canada, and by our counterparts in the study countries, where required. Except in Chile, all participants indicated in writing their consent to participate in the present research; in Chile, participants consented verbally.

### Design.

A mixed methods design^19,20^ combined qualitative data from interviews with key informants who were purposefully selected public health experts, and quantitative data from a CFIR questionnaire.^21^ Data were collected simultaneously with the purpose of seeking complementarity; the “elaboration, enhancement, illustration, and clarification of the results from one method with the results from the other method.”^20^

### Participant characteristics.

Participants included 34 public health experts (19 men and 15 women; Mean age = 57.3 years and standard deviations [SD] = 10.5 years) from eight countries: Chile, India, Pakistan, Bangladesh, Thailand, Vietnam, South Africa, and Nigeria. Participants were identified in each country by the study leads based on their expertise and lifelong careers dedicated to public health (Mean work experience = 29.0 years and SD = 10.3 years). Participants’ roles included advisor or director of health/public health/disease control (*N* = 12), clinician scientist (*N* = 8), laboratory scientist (*N* = 7), researcher (*N* = 3), professor (*N* = 2), and veterinarian (*N* = 2). Demographic characteristics for participants in each country are summarized in Table 1.

**Table 1:** Summary of participant demographic characteristics

	Vietnam (*N* = 5)	Thailand (*N* = 5)	Bangladesh (*N* = 2)	Pakistan (*N* = 4)	Chile (*N* = 4)	India (*N* = 4)	Nigeria (*N* = 6)	South Africa (*N* = 4)*
Gender, *n*								
Males	2	3	1	2	2	2	5	2
Females	3	2	1	2	2	2	1	2
Age in years, M (SD)	51.0 (12.3)	53.8 (11.7)	65.0 (0.0)	59.3 (9.6)	65.8 (14.6)	62.8 (8.8)	55.0 (7.9)	52.3 (3.1)
Education, *n*								
PhD	–	–	1	1	–	–	–	2
MD	1	2	–	2	2	1	–	–
MD, PhD	3	–	–	–	–	2	1	–
MD, MPH	–	1	–	1	–	1	–	–
MD, MSc	1	–	–	–	–	–	–	–
MSc	–	–	–	–	–	–	5	1
MPH	–	1	1	–	–	–	–	–
Doctor in Veterinary Medicine	–	1	–	–	1	–	–	–
Doctor in Veterinary Medicine, MPH	–	–	–	–	1	–	–	–

SD = standard deviations. Age, gender, and education characteristics of interview participants in the eight case countries of interest.

* Note: Demographics data were available for three of the four participants.

### Data collection.

Qualitative data consisted of semi-structured interviews with key informants. With the exception of South Africa, where data were collected via phone interviews, interviews were conducted in person, audio-taped, and transcribed verbatim. With the exception of Chile, all interviews were conducted in English. In Chile, interviews were conducted by local researchers in Spanish, audio-recorded and subsequently translated to English locally. Interviewer training for Bangladesh, Chile, India, and Pakistan involved reviewing the interview tool and was conducted over the telephone. The remaining four countries’ (Nigeria, South Africa, Thailand, and Vietnam) interviews were conducted by Dr. Raluca Barac, a qualitative scientist in our team at The Hospital for Sick Children. The same interview protocol (see Supplemental Appendix A) was followed in each country to explore a predetermined set of typhoid-relevant interventions and factors (i.e., public health campaigns and vaccinations for typhoid, diarrheal disease control, food safety, food handlers, agricultural practices, treatment of sewage, antibiotic medication, and migration). At the same time, informants were also invited to discuss additional interventions or contextual factors they believed to be relevant for their country. The interview protocol was developed based on our conceptual framework^2^ and literature review.

Quantitative data were collected with a CFIR questionnaire which was administered in person by the interviewer following each interview. The CFIR questionnaire, developed by the authors (M.B. and R.B.) for an earlier study,^22^ was used to document specific aspects of the implementation of typhoid-relevant interventions related to intervention characteristics (eight items), inner (12 items) and outer settings (four items), process (eight items), and staff characteristics (five items) for a total of 37 questions. Items were rated on a 5-point Likert scale (1 “very unimportant” and 5 “very important”).

### Data analyses.

Interview data were analyzed using a deductive qualitative content analysis approach, appropriate for exploring and describing lesser known phenomena^23^ such as the implementation of typhoid-relevant interventions in Asia, Africa, and South America. Specifically, analyses mapped onto the two main aims of the present study exploring and describing 1) country-specific typhoid-relevant interventions implemented between 1990 and 2015 and 2) contextual factors perceived to have shaped their implementation.

Content analysis refers to a set of strategies used to analyze textual data in which data are coded and categorized to describe the content and identify common issues, trends, and facts.^23^ We used a deductive approach where codes were determined a priori, based on the CFIR conceptual framework, and data coded against seven large categories of interventions and factors: public health campaigns and vaccinations for typhoid, diarrheal disease control, food safety, food handlers, agricultural practices, treatment of sewage, and antibiotics. Data were analyzed by four authors (A. R., D. A., M. B., and R. B.) working in pairs to ensure that all transcripts were double-coded to achieve qualitative rigor. Data were categorized under the seven predetermined typhoid-relevant interventions, although we remained open to new intervention types that might emerge from the data. Team members worked independently and met regularly to review coding, discuss differences, and make decisions to ensure coding consistency.

Analysis of quantitative data from the CFIR questionnaire was limited by small sample size. As such, summary descriptive statistics are provided for all CFIR domains and constructs to provide a picture of their relative importance.

## RESULTS

Results are presented to reflect the two main aims of the study: 1) describing the typhoid-relevant interventions implemented in the eight countries and 2) exploring the contextual factors associated with their implementation.

### Aim 1: Description of the typhoid-relevant interventions.

**Figure 1.**
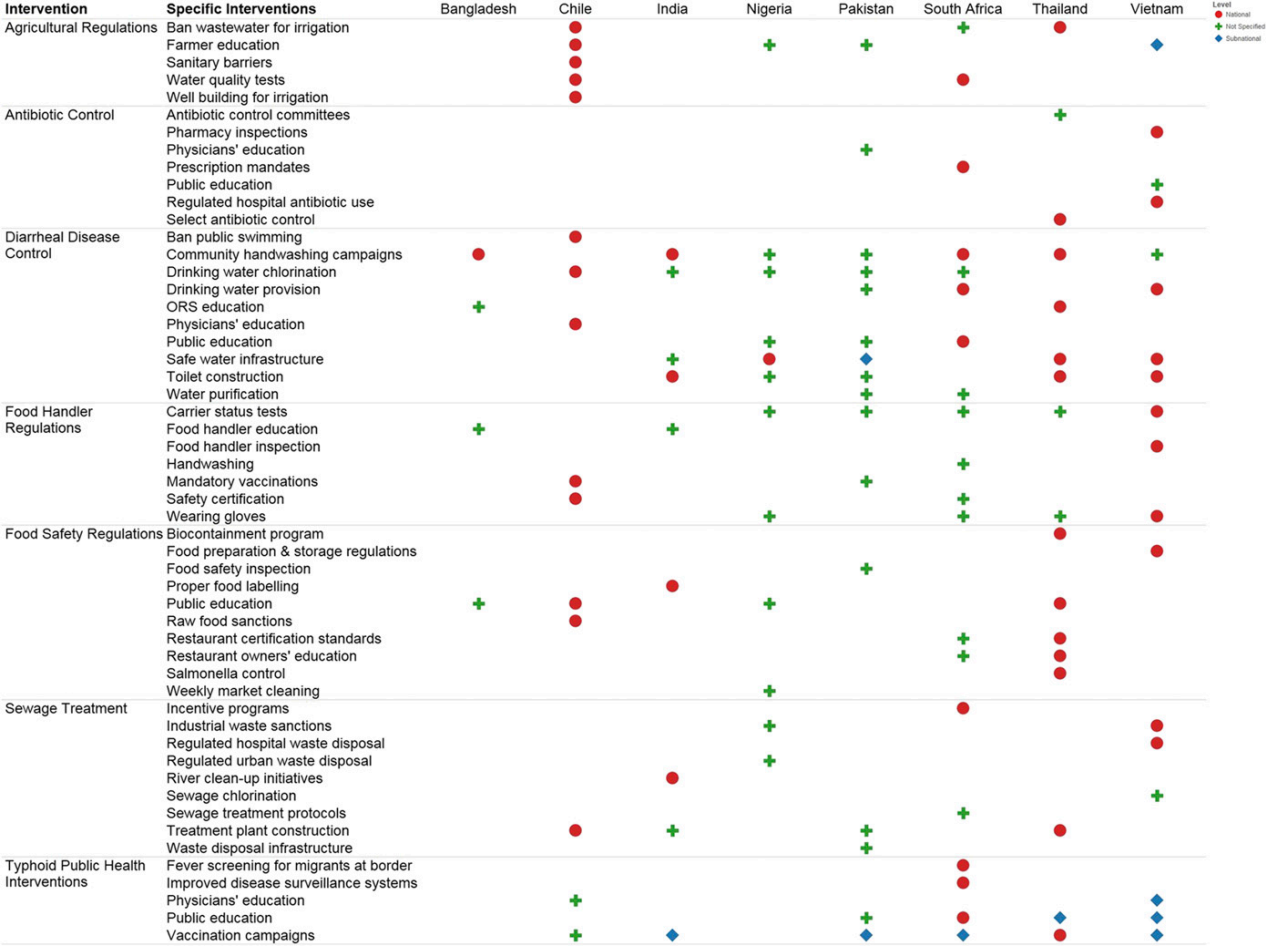
Summary of typhoid-relevant interventions that have been implemented within the eight case countries from 1990 to 2015. This figure outlines the interventions that may have impacted typhoid fever as per in-country interview respondents. The eight intervention categories are listed vertically with specific interventions identified in each country. The eight countries of interest are listed horizontally. Where respondents could identify interventions at the national level, it is depicted with a red circle. Green crosses show interventions where the level of implementation was not specified. Subnational interventions are shown as blue diamonds.

Figure 1 summarizes the seven types of typhoid-relevant interventions implemented in each of the eight countries between 1990 and 2015, along with an indication of the scope of the target population (i.e., national or subnational). Overall, relatively few typhoid-specific interventions were reportedly implemented in these eight countries; however, all countries implemented interventions for diarrheal disease control and regulations for food safety and food handlers. Although most countries implemented agricultural and sewage treatment practices, very few addressed the control of antibiotic medication. No additional control interventions were mentioned by the participants in any of the eight countries, over and above those included in the interview.

This figure summarizes the seven categories of interventions that may have had an effect on the trends in typhoid in Bangladesh, Chile, India, Nigeria, Pakistan, South Africa, Thailand, and Vietnam. Within each of the seven categories, specific interventions are listed, and the presence or absence of these interventions are recorded for each country.

#### Typhoid-specific interventions.

Typhoid-specific interventions typically occurred in response to local typhoid outbreaks and included vaccination campaigns; public education regarding the causes, treatment, and prevention of typhoid fever; and physician education to improve diagnosis and treatment. In Vietnam, for instance, typhoid fever outbreaks in the Mekong River Delta in the early 1990s triggered vaccination programs in the area and public education about proper handwashing techniques and boiling water before consumption. Typhoid-relevant interventions in Pakistan were implemented in response to emergency situations, such as the earthquake in 2005 and floods in 2010, and focused on vaccinations for children younger than 5 years living in the affected areas and public education on clean water and sanitation. Typhoid control in South Africa was similar, such that local typhoid outbreaks stimulated improvements in water systems, vaccination campaigns, and fever screening for migrants at the borders. Not all countries reportedly implemented interventions specifically targeting typhoid fever (i.e., Nigeria and Bangladesh reported no typhoid-specific interventions between 1990 and 2015).

#### Diarrheal disease control.

Interventions for diarrheal disease control were implemented in all countries through a variety of activities, including public health campaigns for handwashing; building, improving, or expanding the safe water infrastructure; and building toilets. In Thailand, efforts to expand the pipe water system across the country spanned 30 years (1970s to 2000) and latrine construction occurred over four decades, ending in the 2000s. Community health volunteers played a key role in these initiatives and communities who accomplished the task were recognized with a golden latrine or jar symbol. In Nigeria, handwashing campaigns promoting the slogan “your life is your hands” intensified in response to the country’s 2014 Ebola epidemic. Messages about handwashing and drinking clean water were disseminated via multiple communication channels, including talks given at schools, market places, churches, and mosques; jingles on the radio; announcements in schools; advertisements in markets and bus stops; and participation in the Global Hand Washing Day. Although fear of Ebola was a strong motivator for behavior change and placed control measures as a high priority for the country, Nigeria’s promotion of handwashing and drinking safe water predates this crisis and has more longstanding history, as illustrated by a large school campaign implemented in 2008 that reached 1.5 million children and a United Nations Children’s Fund-led campaign that provided motorized borehole pumps as early as 2000. In South Africa, typhoid outbreaks in 2000s were commonly traced back to water contamination that triggered significant investments in improving access to safe water and sanitation and public education.

#### Food safety regulations.

All eight countries implemented interventions to regulate food safety. Public education interventions on food safety took place in four countries (Chile, Nigeria, Bangladesh, and Thailand), whereas interventions to regulate the quality of street and restaurant food were conducted in Chile, Nigeria, Pakistan, South Africa, Thailand, and Vietnam (note that in Bangladesh, food regulations exist but have not been implemented). In response to the first cholera cases in the 1990s, Chile introduced a public education campaign for cooked food and banned restaurants from serving raw foods. Thailand implemented multiple food safety interventions from 1990 to 2015, beginning with a “National Salmonella Control Program.” In addition, a “Biocontainment Program” worked to ensure that food produced in Thailand met European Union food safety standards for export. The “Farm to Table” and “Clean Food, Good Taste” campaigns targeted food quality and safety by ensuring that restaurants met certain criteria to receive the “Clean Food, Good Taste” certificate and by educating the public to only eat at restaurants with this certification. In Nigeria, mandatory regulation coupled with consistent inspections and fines ensure that market stalls are cleaned every Thursday before market opening.

Not all food safety interventions have been implemented effectively. Efforts to ban street food in metropolitan regions in India failed, but more recent initiatives aimed at improving food standards and food labeling have been more successful. Similarly, in Vietnam and South Africa, regulations for food storage, washing, and preparation are in place, but follow-up to ensure effective implementation and adherence are variable, with large differences emerging between rural and urban areas. Inconsistent follow-up and inspection of food safety standards is also an issue for Pakistan.

#### Food handler regulations.

Regulations related to food handlers varied among study countries. In India and Bangladesh, interventions targeting food handlers focused on food safety education. The other six countries implemented mandatory vaccinations and/or tests to check carrier status for disease. Thailand has implemented European Union regulations in food factories, including biannual mandatory health checks for workers in chicken factories and annually for workers in seafood factories. Similarly, food handlers diagnosed with typhoid fever in South Africa are required to stop working until three consecutive stool samples are found to be free of *Salmonella* Typhi. Measures to ensure implementation and adherence of food handler regulations occur variably across the study countries and are fraught with adherence issues. Vietnam conducts random checks on food handlers, and Nigeria, Thailand, and Vietnam promote gloves for food handlers. All interviewees noted that limited surveillance was a barrier to the successful implementation of food handler regulations.

#### Agricultural practices.

Interviewees from India and Bangladesh noted there are no specific measures in place to regulate agricultural practices related to typhoid control. By comparison, Chile, Nigeria, Pakistan, and Vietnam commonly provide farmer education with a focus on safe irrigation of crops and discontinuing the practice of using wastewater for this purpose. In Thailand, similar measures preventing the use of wastewater for irrigation have been in place before 1990s. The use of wastewater for irrigation and tests of water quality were also implemented in South Africa. Chile has had a strong emphasis on the regulation of agricultural practices from 1990 to 2015, driven by the identification of the first cases of cholera in early 1990s. Although the use of wastewater for crop irrigation was banned by a decree passed in 1967, the implementation of these regulations only began in the 1980s and was only drastically enforced in the 1990s to control the spread of cholera. Several behavior change strategies were implemented, including fines for farmers who used wastewater for irrigation, destruction of crops that showed contamination or were irrigated with wastewater, mandatory building of wells for irrigation, mandatory testing of the irrigation water, education of students in agriculture and related programs regarding water contamination and safe irrigation, and sanitary barriers preventing the contaminated crops from being spread to the whole country.

#### Sewage treatment.

With the exception of Bangladesh, where interviewees were unaware of any specific sewage treatment interventions implemented between 1990 and 2015, the remaining seven countries noted progress in the last 25 years, most notably with the construction of treatment plants for sewage (Chile, Pakistan, and India), establishing incentives for improving sewage treatment quality and sanctions on industrial, urban, or hospital waste (South Africa, Nigeria, and Vietnam), improving the waste disposal infrastructure (Pakistan), cleaning up major rivers (India), and continuing the implementation of older regulations for treatment sewage (Thailand).

#### Antibiotic control.

Most of the study countries undertook few, if any, measures to control the sale and use of antibiotic medication, although all interviewees noted the issue of rising rates for antimicrobial resistance. The exception is South Africa, as the sale of antibiotics here is strictly controlled. Some countries made efforts to educate physicians regarding antimicrobial resistance (Pakistan) or to initiate public education campaigns regarding the risks of taking antibiotics without prescription (i.e., “Antibiotic Awareness Week”), to regulate antibiotic use in hospitals, and conduct random pharmacy audits by the Ministry of Health (Vietnam). In Thailand, most of the antibiotics are sold over the counter with controls for only select few.

#### Perceived intervention effectiveness.

**Figure 2.**
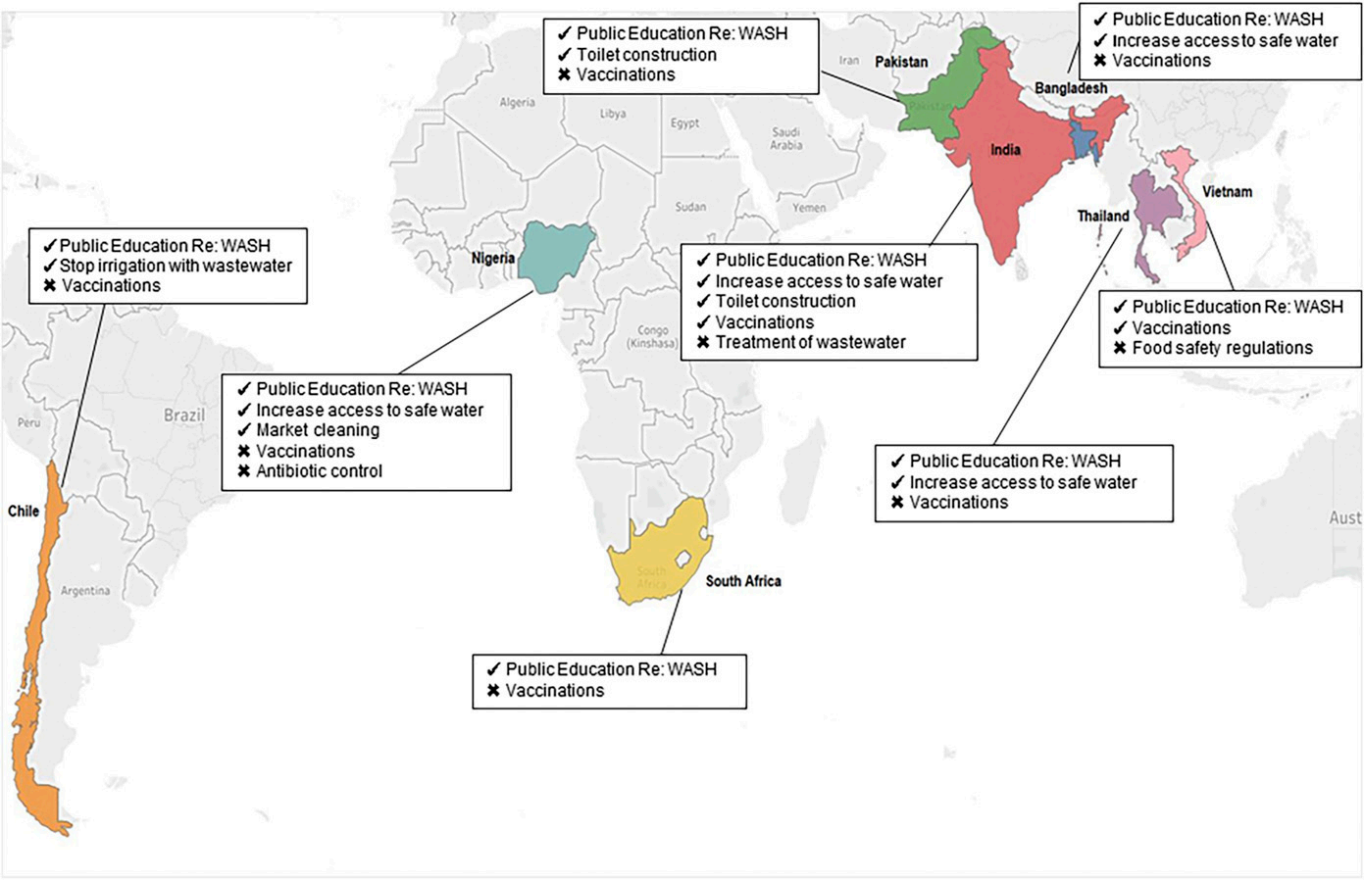
Perceived most and least effective typhoid-relevant interventions implemented in eight countries between 1990 and 2015 based on interview data. This figure highlights the eight case countries on a map with the typhoid-relevant interventions identified by interview participants as being the most and least effective for typhoid control.

Figure 2 captures participants’ reflections on effectiveness of typhoid-relevant interventions implemented in their countries over the last 25 years. There is remarkable consistency both within- and between-countries, with all eight countries noting public education regarding WASH as the most effective intervention. In addition, five interventions were perceived to have been effective for typhoid control in some but not all eight countries: increasing access to safe water (in four countries), toilet construction (in two countries), vaccinations (in two countries), market cleaning (in one country), and banning of wastewater for irrigation (in one country).

This figure presents the typhoid-relevant interventions that interview participants in each case country deemed most effective, denoted by a checkmark and least effective denoted in the figure by an “x” in the control of typhoid fever.

Interviewees from six of the eight countries noted vaccination campaigns as the least effective intervention because of low cost-effectiveness, negative side effects (particularly for the earlier vaccine versions), low awareness in the population regarding the existence of typhoid vaccines, and the general belief that vaccines alone could not control typhoid in the absence of additional interventions, such as WASH. Antibiotic control campaigns (Nigeria), treatment of wastewater (India), and food safety regulations (Vietnam) were also perceived as ineffective in controlling typhoid, mainly for reasons related to poor implementation.

### Aim 2: Description of the contextual factors.

Table 2 summarizes the means and SD for CFIR domains, and within each domain, for the construct with the highest ratings in each of the eight countries. The average ratings for the five CFIR domains were centered around and above the middle point of the scale. The same constructs were consistently rated as highly associated with implementation success across the eight countries: Intervention Characteristics 1) evidence strength and quality, 2) relative advantage, and 3) adaptability; Outer Setting 1) patient needs and resources and 2) external policy and incentives; Inner Setting 1) organizational incentives and rewards and 2) available resources; Staff Characteristics 1) knowledge and beliefs about the intervention and 2) self-efficacy; Process 1) planning, 2) engaging, 3) formally appointed implementation leaders, and 4) reflecting and evaluating.

**Table 2:** Descriptive statistics (M [SD]) for the five CFIR domains and the constructs with the highest ratings, by country*

	Vietnam	Thailand	Bangladesh†	Pakistan	India	Nigeria	South Africa
Intervention characteristics	4.00 (0.44)	3.55 (0.62)	4.88 (0.18)	3.70 (0.59)	4.06 (0.24)	3.58 (0.36)	4.00 (0.76)
Evidence strength and quality	–	–	5.00 (0.00)	4.00 (0.82)	4.50 (0.58)	4.67 (0.52)	–
Relative advantage	–	4.80 (0.45)	5.00 (0.00)	4.00 (0.00)	–	4.67 (52)	–
Adaptability	4.80 (0.45)	4.80 (0.45)	–	–	4.50 (0.58)	4.67 (0.52)	4.67 (0.58)
Outer setting	3.90 (0.38)	3.05 (0.48)	2.25 (–)	3.81 (0.75)	3.25 (0.54)	3.21 (0.25)	3.25 (1.06)
Patient needs and resources	4.80 (0.45)	4.60 (0.89)	4.00 (–)	–	4.50 (0.58)	4.67 (0.52)	3.50 (2.12)
External policy and incentives	–	–	–	4.00 (0.82)	–	–	3.50 (0.71)
Inner setting	4.27 (0.36)	3.90 (0.11)	3.71 (0.65)	3.46 (0.55)	3.38 (0.43)	3.93 (0.23)	3.32 (0.92)
Organizational incentives and rewards	–	–	4.00 (1.41)	4.25 (0.50)	–	–	–
Available resources	4.40 (0.89)	4.60 (0.55)	5.00 (–)	–	4.25 (0.96)	4.83 (0.41)	4.00 (1.73)
Staff characteristics	4.04 (0.30)	2.96 (0.61)	3.00 (–)	3.15 (0.93)	4.15 (0.68)	3.00 (0.91)	3.33 (0.81)
Knowledge and beliefs about the intervention	4.60 (0.55)	4.00 (0.71)	4.00 (–)	–	–	4.50 (0.84)	–
Self-efficacy	–	–	4.00 (–)	3.50 (1.00)	4.50 (0.58)	–	4.00 (1.73)
Process	3.85 (0.24)	3.70 (0.55)	4.00 (–)	3.47 (0.53)	4.06 (0.77)	3.52 (0.62)	3.92 (1.23)
Planning	–	4.80 (0.45)	5.00 (–)	–	–	4.67 (0.52)	–
Engaging	–	–	5.00 (–)	–	4.50 (0.58)	–	–
Formally appointed implementation leaders	–	–	5.00 (–)	4.25 (0.96)	–	–	4.33 (1.15)
Reflecting and evaluating	4.80 (0.45)	–	5.00 (–)	–	–	4.67 (0.52)	4.33 (1.15)

CFIR = consolidated framework for implementation research; SD = standard deviations. A summary of the highest rated constructs within the five CFIR domains (intervention characteristics, outer setting, inner setting, staff characteristics, and process), broken down by country. An average score is reported for the domain and highest rated construct with a SD presented in brackets.

* Note: Due to logistical constraints, no CFIR data were collected for Chile.

† CFIR data from Bangladesh is based on the responses of two interviewees.

In addition, qualitative analyses highlighted several contextual factors perceived to have influenced the implementation of typhoid-relevant interventions as enablers (economic development, the use of multiple implementation strategies, other epidemics or outbreaks, changes in government administration, and the pressure of hosting an international event) or barriers (limited resources and planning, habitual behaviors and cultural practices, and migration) (see Table 3). Most barriers and some of the enabling factors (i.e., economic development and the use of multiple implementation strategies) were commonly noted across countries, although some enabling factors were unique to a specific country (e.g., changes in government administration in Thailand, the pressure of hosting an international event in Nigeria, and other epidemics or outbreaks in Nigeria and Chile).

**Table 3:** Summary of contextual factors influencing the implementation of the typhoid control interventions

Factor	Description
Economic development	The role of economic development was noted in Chile, Bangladesh, South Africa, Vietnam, and Thailand in facilitating the implementation of typhoid interventions by improving living conditions, strengthening the water and sanitation infrastructure, and increasing literacy levels. By example, interviewees in Thailand believe that economic development was stimulated by monies flowing into the country from Thai people working in the Middle East (1990s to 2000) and by economic specialization that started in the 1990s (i.e., each region was asked to identify a service or product to become symbolic for that region and to work toward excelling at it)
The use of multiple implementation strategies	Interviewees from most of the study countries discussed the importance of using multiple strategies to target behavior change, such as the use of television advertisements, radio messages, pamphlets, market and church announcements, and school campaigns to promote handwashing or food safety. Multiple implementation strategies were thought to contribute to the success of public education/behavior change efforts by both validating the message and increasing the population reach
Tension for change created by the onset of other epidemics or outbreaks	Interviewees highlighted fear of Ebola (Nigeria, 2014) and cholera (Chile, 1991) as strong motivational factors for behavior change with respect to water and sanitation, food, and agricultural practices. These epidemics/outbreaks created a tension for change (Consolidated Framework for Implementation Research outer setting factor) and contributed to reductions in typhoid incidence. Interviewees emphasized that these diseases were crucial to generating motivation for change. For instance, cholera was perceived as a “diabolic” or “killer” disease, whose disastrous effects in Peru motivated people to implement drastic changes in Chile (destroying crops). Paradoxically, typhoid fever was endemic to Chile and people were habituated to it, and when typhoid rates doubled in 1967, no measures were taken to control the spread of the disease.
Changes in government administration	In Thailand, the decentralization of power (1997) contributed significantly to implementation efficiency for typhoid control. Specifically, changes in government structure resulted in the delegation of power to local authorities and empowerment of local communities. In practice, this meant that communities could maintain effective control and take action on food safety, regulation of markets, sanitation, and water supply instead of waiting for government action. Similarly, South Africa becoming a democracy in 1994 led to changes such as more equitable distribution of resources, better quality, and access to housing and medical care
The pressure of hosting of an international event	In Nigeria, for instance, the pressure of hosting the Fédération Internationale de Football Association (FIFA) World Youth Championship in 1999 was identified by interviewees as a key factor stimulating the implementation of regulations for food safety and food handlers. Implementation efforts were driven by a need to encourage FIFA delegates and visitors and to shake the perception that visiting Nigeria put them at risk of contracting cholera
Limited resources and planning	Participants in all countries discussed how limited resources and planning are barriers to the effective implementation and sustainability of typhoid control interventions. This included insufficiency of staff for monitoring whether regulations for food safety, and food handlers were actually implemented on the ground and with what degree of fidelity or compliance, and limitations imposed by the lack of data monitoring implementation efforts. Such common implementation barriers highlight the difficulties of achieving good outcomes with effective interventions that may be poorly implemented
Habitual behaviors and cultural practices	Resistance to change and the power of habitual behaviors (e.g., open defecation, drinking water without purifying or boiling it, eating raw food such as ceviche in Chile and raw blood soup in Vietnam, and using wastewater for irrigation) were noted in all countries as barriers to the implementation of typhoid control interventions and were typically addressed through public education
Migration	Interviewees from all study countries discussed population migration within country (rural to urban in Bangladesh, India, and Chile; from north to south in Nigeria and Vietnam; and related to disasters and conflicts in Nigeria and Pakistan) and from neighboring countries (Nigeria, Chile, and Thailand), but no linkages were made to changes in the rates of typhoid fever. Interviewees from Nigeria, Pakistan, and Vietnam, however, believed that population migration contributed to the spread of typhoid fever, especially in the refugee camps, although there were no data to support this belief

The eight contextual factors that were identified to have played a role in the implementation of typhoid fever control interventions. Descriptions are provided for each contextual factor using respondent data across the eight case countries.

## DISCUSSION

Past research has focused on the monitoring of typhoid rates with little attention to implementation methods or effectiveness of typhoid control interventions. To address this gap, the present study purposefully sampled key informants working in public health to explore a range of typhoid-relevant interventions implemented in eight countries: Chile, India, Pakistan, Bangladesh, Thailand, Vietnam, South Africa, and Nigeria. Our aims were to scope 1) the typhoid-relevant interventions implemented in these countries between 1990 and 2015 and 2) the contextual factors perceived to be associated with their implementation, based on a determinant implementation model^24^ called the CFIR.^25^ The study findings provide a snapshot of typhoid-relevant interventions implemented over 25 years and the factors considered to be associated with implementation success from the perspective of a small sample of key informants working in public health in these endemic countries. These findings have the potential to inform future systematic investigations of the implementation of typhoid control interventions and LMIC health evidence implementation more broadly (e.g., methods and effectiveness of implementation) and contribute to a better understanding of how such implementation efforts impact typhoid rates.

Overall, with the exception of South Africa, relatively few typhoid-specific interventions and measures to control the use of antibiotic medication were reportedly implemented in these eight countries. A concern in the lack of control surrounding antibiotic use lies in the diagnosis of typhoid fever and the resultant increase in antibiotic resistance. The availability and quality of diagnostic tests for infectious diseases vary widely in LMICs.^26^ Within certain contexts, the capacity to perform culture tests to confirm infectious pathogens is not present either because of a lack of trained personnel or diagnostic tools.^26^ In other settings, available tests fall short of the gold standard.^26^ For example, Widal tests are used in resource-limited environments; however, it lacks the necessary sensitivity and specificity to accurately diagnose typhoid and paratyphoid fever.^27^ This can lead to overestimation of the incidence of typhoid, and in turn, inappropriate prescription of antibiotics.^27,28^ An assessment of multidrug-resistant (MDR) *S.* Typhi strains, characterized as being resistant to ampicillin, chloramphenicol, and co-trimoxazole, noted that MDR has been on the rise since the 1980s.^29^ A study conducted in Cameroon, reviewed how medical professionals were interpreting Widal tests and assigning treatment to patients.^28^ This study found that within their sample, 84% of nurses and just less than 50% of doctors had difficulty identifying those who did not need treatment, which in turn led them to prescribe treatment that was unnecessary.^28^ The over prescription of antibiotics has played a key role in the increasing burden of drug and MDR strains of *S.* Typhi being observed in sub-Saharan Africa and South Asia and remains a major public health concern.^26,28^

However, most of the study countries implemented agricultural and sewage treatment practices and all study countries implemented interventions for diarrheal disease control and regulations for food safety and food handlers. The relationship between outbreaks and carrier interventions was not specifically captured, but it was noted in five countries (Nigeria, Pakistan, South Africa, Thailand, and Vietnam) that carrier status testing was performed in food handlers. Vietnam was the only country where respondents confirmed the testing of food handlers for typhoid carriage occurred at the national level, the level of implementation in the remaining four countries was unspecified (Figure 1). Interventions for diarrheal disease control, in particular, public education regarding WASH, were perceived to be the most effective measures for typhoid control by most key informants across the eight countries, whereas typhoid vaccinations were perceived as the least effective typhoid control intervention in six of the eight countries.

These data provide a descriptive snapshot of the strategies implemented for typhoid control across the eight countries over a period of 25 years and suggests future implementation directions. What remains unknown are the specific implementation and facilitation methods that were followed to implement these strategies and whether typhoid control interventions were implemented with high compliance and/or fidelity. In other words, we have no indication of implementation outcomes as distinct from service system outcomes and clinical treatment outcomes.^26,27^ Implementation outcomes are the effects of deliberate and purposive actions to implement new treatments, practices, and services, and they have three important functions: 1) they serve as indicators of the implementation success, 2) they are proximal indicators of implementation processes, and 3) they are key intermediate outcomes^29,30^ in relation to service system or clinical outcomes in treatment effectiveness and quality-of-care research. Implementation outcomes serve as necessary preconditions for attaining desired changes in health, clinical, or service outcomes because interventions cannot be effective if they are poorly implemented.^3^

Analyses of contextual factors that are important for implementation success highlighted several factors associated with implementation success across the eight countries: Intervention Characteristics, including 1) the evidence strength and quality of the health intervention, 2) its adaptability, and 3) its relative advantage over other health interventions; Outer Setting Characteristics such as 1) the population needs and resources and 2) external policies and incentives; Key Inner Setting Characteristics included 1) the presence of organizational incentives and rewards and 2) available resources; Staff Characteristics perceived as associated with effective implementation of typhoid controls included 1) staff with solid knowledge and beliefs about the intervention and 2) a sense of self-efficacy; Process Characteristics identified as important included 1) planning for change, 2) engaging key people in the implementation, 3) the presence of formal implementation leaders, and 4) the opportunity to evaluate and reflect on the implementation process and outcomes.

These findings are remarkably consistent with past research that has explored CFIR contextual factors associated with implementation success in different sectors (health, global health, and mental health) and settings (LMICs).^4,21,22,25,31,32^ Taken together, these studies highlight a subset of CFIR contextual factors identified by key informants as more highly associated with implementation effectiveness. Knowing which factors are more “important” relative to effective implementation has implications for advancing implementation knowledge and practice in global health, such that factors that emerge as highly associated with success can be taken into account in implementation planning, monitoring, and evaluation.

A number of additional contextual factors emerged as enablers (economic development, the use of multiple implementation strategies, other epidemics or outbreaks, changes in government administration, and the pressure of hosting an international event) or barriers (limited resources and planning, habitual behaviors and cultural practices, and migration) to the implementation of typhoid-relevant interventions in these countries. Some of these were common across countries (e.g., economic development), whereas others were country-specific (e.g., other epidemic or outbreaks). Some of the barriers identified in the present study aligned with those noted in a review by Puchalski Ritchie et al.^7^ (e.g., limited human and financial resources). In addition, interviewees in most of the study countries discussed the importance of implementation quality and adherence to the interventions implemented, noting that the adoption of evidence-based interventions was necessary but insufficient without comparative effort in the monitoring of implementation process and intervention adherence.

### Limitations.

Several limitations to the present study are noted. First, the number of key informants from each of the eight countries was relatively small, and this limited analyses of questionnaire data. Second, the study design would have been strengthened by document analysis to confirm and supplement the information provided by the key informants relative to the interventions they discussed. In the absence of validation from external documents, we rely solely on key informant recollections of typhoid control events over a 25 years period. Relatedly, we note that because of the small sample size across countries, the public health expertise of our key informants may not have been inclusive of all types of typhoid-relevant interventions implemented in their country, and thus, it is likely that interventions other than the ones identified in the present study were implemented either nationally or subnationally. Finally, there are insufficient data to allow reliable linkage of typhoid-relevant interventions and contextual implementation factors with actual changes in typhoid rates. This level of analysis would have required country-level typhoid surveillance data, which are not available, and external documentation of dates and types of interventions implemented within each country over this 25-year period. Despite these limitations, our study findings provide an important starting point for future systematic investigations of typhoid control interventions and implementation of evidence-based health interventions in LMICs and endemic regions, and contribute to a body of research evidence seeking to validate and refine the CFIR.

## Supplementary Material

Supplemental appendix
