# Supplementary material for: Implementation of Interventions for the Control of Typhoid Fever in Low- and Middle-Income Countries
Source: Am J Trop Med Hyg. 2018 Jul 25;99(3 Suppl):79–88. doi: 10.4269/ajtmh.18-0110 (PMC6128369; doi:10.4269/ajtmh.18-0110)
Supplement: Supplementary file 1 [file tpmd180110.SD1.pdf]

Tackling Typhoid - What do Global and Country Trends Teach Us? A Prospective Study:
Interview Protocol

Date: ________________________________ **Time of the Interview**: ___________________________

Interviewer: ___________________________ **Interviewee Study ID number**: _______________________________

Interview Questions

We are doing a study looking at trends in typhoid in eight countries since 1990. [Country name] is one of the eight countries we are interested in. We examined the numbers and now we are at a stage where we are trying to understand the factors important for typhoid control – why and how changes occurred over the last 25 years. We wanted to talk to you because of your valuable knowledge and expertise in this area. You are here because you consented to a one-on-one interview about enteric fever trends in [Country name]. During this interview, you will be asked to provide information about you (state experiences, personal opinions, etc) which may be unique to you, the organizations you work/worked with and the country you (live in/work with, etc). This means that you may potentially be identifiable based on the information you provide. This interview will be audio recorded to allow us to document information more accurately. You are at liberty to withdraw from this interview at any point.

- For the beginning, please tell us about your overall work experience related to typhoid control.
    - *If interviewee was not directly involved in any initiative, encourage them to talk about their knowledge of typhoid*.

- To start talking about typhoid fever, please think about the last 25 years and draw a line on this paper to show how you think the rates of typhoid have changed in [country name] across time, since 1990 until present. We know that in the last 25 years the rates of typhoid have changed in your country but we would like to understand what that might have looked like by drawing a line on this page so that you can show us when, how and why those changes took place from your perspective. You can draw a line on this page and in any way you think best shows your point of view about how those changes took place. Please also add any details about the intervention or what was happening at that time that had an impact on the intervention as it was introduced to the community *[The interviewee draws the trajectory]*

- Now, looking at this, please identify the interventions for typhoid control used in your country since 1990. *[The interviewee marks these interventions and their associated timelines on the paper; the interviewer marks them on the list below to follow up with more specific questions.]*
    - How involved were you in these interventions?

- Let's look more specifically at the interventions you mentioned [*most likely the interventions they mention overlap with the list below, in which case follow up with the questions listed for each intervention, as in Q2-Q10; if it is a new intervention, follow up on each of the new factors mentioned by the interviewee as in Q1. For all interventions discussed, make sure that the interviewee gives information about location and timing and enough detail about the intervention to have clarity about what exactly was done*]

Q1	You mentioned that **[new intervention mentioned by the interviewee**] was an important intervention for typhoid control.	Tell me more about what it involved in your country.<br>When did the intervention take place?<br>Where did it take place?<br>How many people did it reach? [*get information about initiation and scale-up]*

Study ID number: ______________________________

		How was it implemented /done? How well was it implemented? Were there any ways to ensure that the intervention was implemented well? What were the main barriers and facilitators?<br><br>How well did it work overall? / What can you tell me about the success of this intervention? / What makes you say this, what kind of evidence do you have? / If it did not work, was it most likely because of the way it was implemented, or the intervention itself?
Q2	You also mentioned **public health promotion campaigns related to typhoid**.	Tell me more about this.<br>When did the intervention take place?<br>Where did it take place?<br>What exactly did it involve?<br>How many people did it reach? [*get information about initiation and scale-up]*<br><br>How was it implemented /done? How well was it implemented? Were there any ways to ensure that the intervention was implemented well? What were the main barriers and facilitators?<br><br>How well did it work overall? / What can you tell me about the success of this intervention? / What makes you say this, what kind of evidence do you have? / If it did not work, was it most likely because of the way it was implemented, or the intervention itself?
Q3	**Diarrheal disease control**	Tell me more about what any campaigns that were carried out to control diarrhea? [*ask specifically about availability of bottled water and behavioral strategies such as changes in washing hand behaviors*]<br>When did the intervention take place?<br>Where did it take place?<br>What exactly did it involve?<br>How many people did it reach? [*get information about initiation and scale-up]*<br><br>How was it implemented /done? How well was it implemented? Were there any ways to ensure that the intervention was implemented well? What were the main barriers and facilitators?<br><br>How well did it work overall? / What can you tell me about the success of this intervention? / What makes you say this, what kind of evidence do you have? / If it did not work, was it most likely because of the way it was implemented, or the intervention itself?
Q4	Another intervention you referred to was **food safety policies and campaigns** put in place since 1990	Tell me more about this – what specific food safety policies and campaigns were put in place?<br>When did the intervention take place?<br>Where did it take place?

	to reduce risk of foodborne infections.	How many people did it reach? [*get information about initiation and scale-up]*<br><br>How was it implemented /done? How well was it implemented? Were there any ways to ensure that the intervention was implemented well? What were the main barriers and facilitators?<br><br>How well did it work overall? / What can you tell me about the success of this intervention? / What makes you say this, what kind of evidence do you have? / If it did not work, was it most likely because of the way it was implemented, or the intervention itself?
Q5	You also mentioned **public health interventions involving food handlers**.	Tell me more about this – what exactly did it involve?<br>When did the intervention take place?<br>Where did it take place?<br>How many people did it reach?<br>How was it implemented /done? How well was it implemented? Were there any ways to ensure that the intervention was implemented well? What were the main barriers and facilitators?<br><br>How well did it work overall? / What can you tell me about the success of this intervention? / What makes you say this, what kind of evidence do you have? / If it did not work, was it most likely because of the way it was implemented, or the intervention itself?
Q6	**Agricultural practices** are another kind of intervention used for typhoid control.	Tell me more about this – What policies have been put in place to prevent irrigation of crops with wastewater?<br>When did the intervention take place?<br>Where did it take place?<br>How many people did it reach? [*get information about initiation and scale-up]*<br><br>How was it implemented /done? How well was it implemented? Were there any ways to ensure that the intervention was implemented well? What were the main barriers and facilitators?<br><br>How well did it work overall? / What can you tell me about the success of this intervention? / What makes you say this, what kind of evidence do you have? / If it did not work, was it most

		likely because of the way it was implemented, or the intervention itself?
Q7	**Treatment of sewage/wastewater** is another kind of intervention used for typhoid control.	Tell me more about this practice – what exactly did this involve in your country?<br>Is sewage treated before being released into the dumping body?<br>When did the intervention take place?<br>Where did it take place?<br>How many people did it reach? [*get information about initiation and scale-up]*<br><br>How was it implemented /done? How well was it implemented? Were there any ways to ensure that the intervention was implemented well? What were the main barriers and facilitators?<br><br>How well did it work overall? / What can you tell me about the success of this intervention? / What makes you say this, what kind of evidence do you have? / If it did not work, was it most likely because of the way it was implemented, or the intervention itself?
Q8	Let's talk about **antibiotic medication** used for controlling typhoid.	What can you tell me about this in your country based on antibiotics sales or use data since 1990?<br>What can you tell me about annual or monthly data on antibiotic prescriptions or sales?<br><br>How was it implemented /done? How well was it implemented? Were there any ways to ensure that the intervention was implemented well? What were the main barriers and facilitators?<br><br>How well did it work overall? / What can you tell me about the success of this intervention? / What makes you say this, what kind of evidence do you have? / If it did not work, was it most likely because of the way it was implemented, or the intervention itself?
Q9	**Vaccination campaigns** have also been used to control typhoid.	Tell me more about this in your country since 1990.<br>What types of vaccines were used?<br>What population was targeted?<br>How was this population targeted?<br>Where and when did these campaigns happen?<br>What do you know about the number of people vaccinated? [*get information about initiation and scale-up]*

		How was it implemented /done? How well was it implemented? Were there any ways to ensure that the intervention was implemented well? What were the main barriers and facilitators?<br><br>How well did it work overall? / What can you tell me about the success of this intervention? / What makes you say this, what kind of evidence do you have? / If it did not work, was it most likely because of the way it was implemented, or the intervention itself?
Q10	**Migration events/ migrant workers** and internal displacement are also related to typhoid.	Do you know of any data on internal migration since 1990 - either from town to town or state to state - that could have been related to typhoid changes? The influence of migrant workers, for example?<br>What are the locations involved?<br>When did this happen?<br>Roughly, how many people migrated?

- We are getting close to the end of the interview. So far, we talked about several interventions used in [country name] since 1990 and the information you gave me is very helpful. Thinking of all the things we discussed – public health typhoid campaigns, agricultural practices, vaccination campaigns, antibiotics, treatment of wastewater, food safety policies, migrant workers – what do you think were the most effective interventions for typhoid control in your country since 1990? What about the least effective interventions?
    - What made you say that this invention was the most or least effective?

- Finally, looking to the future, what are your thoughts or recommendations about controlling typhoid in your country?

Study ID number: ______________________________

Sample and Inclusion Criteria

We are targeting people who are knowledgeable about typhoid, i.e., key informants on the topic, about 4-5 interviewees in each of the eight countries examined. We will include people who:

- are knowledgeable about/ have been involved in typhoid control work in their respective countries;
- ideally, they are able to speak English (but this is not an exclusion criterion);
- are willing to participate in an interview conducted in person to talk about their work experience with typhoid;
